# Expect Nothing: The (Lack of) Influence of Subjective Life Expectancy on Valuation of Child Health States

**DOI:** 10.3389/frhs.2022.803109

**Published:** 2022-04-04

**Authors:** Stefan A. Lipman

**Affiliations:** Department of Health Economics, Erasmus School of Health Policy & Management, Erasmus University Rotterdam, Rotterdam, Netherlands

**Keywords:** subjective life expectancy, EQ-5D-Y, proxy perspective, time trade-off, child health

## Abstract

**Objective:**

Earlier research has shown that individuals' subjective life expectancy (SLE) affects health state valuation with time trade-off (TTO). Individuals with longer expected life durations are less willing to trade-off life duration, which yields higher utilities. In this article, the influence of SLE is explored in the valuation of EQ-5D-Y-3L with a proxy perspective, i.e., adults' valuation of health states considering the life of a 10-year-old child. As SLE for children is likely higher, this might explain earlier findings suggesting that individuals are less willing to trade-off years of life for children than for adults.

**Methods:**

A total of 197 respondents were recruited to take part in digital TTO interviews, facilitated by trained interviewers. TTO interviews were implemented in accordance with the recommended protocol for the valuation of EQ-5D-Y-3L. Respondents valued 10 EQ-5D-Y-3L health states for a 10-year-old child, after which they were asked to report how old they themselves expected to become and also how old they expected a 10-year-old child to become.

**Results:**

Generally, adult respondents reported higher SLE for children than for themselves. Neither SLE was systematically associated with the willingness to trade lifetime or the number of life years traded off in TTO tasks. This null-result was substantiated by regression analyses per health state.

**Conclusion:**

The results of this study suggest that individuals' expectations about longevity are not associated with EQ-5D-Y-3L valuation. This lack of association is in contrast to earlier work and might be explained by the psychological distance introduced with proxy perspective valuation, or by the methodological differences with earlier work.

## Introduction

Allocation and reimbursement decisions in health are often informed by economic evaluation, in which the costs associated with treatment-related health gains are compared with the health benefits of treatment. In cost-utility analyses, these health benefits are typically expressed as quality-adjusted life-years (QALYs), which (in principle) enables comparing health benefits across many different treatments and populations (1). Such comparisons may be complex, because the length of life and quality of life gained through treatment may differ substantially. In that context, QALYs are calculated by multiplying the duration of health gains by a weight reflecting the utility of the health status experienced. These utilities are normalized, such that 1 and 0 reflect the “value” of perfect health and being dead, respectively, with negative weights associated with states worse than dead.

Although various methods can be used for measuring these health utilities, many cost-utility analyses rely on health utility measurement (partially) based on the time trade-off (TTO) method (2). In TTO, individuals are asked to imagine a life in impaired health and asked how many years in full health they would find equivalent to it. Hence, in TTO respondents are asked to trade-off between the two dimensions that comprises QALYs, and their trade-offs allow inferring the utility associated with impaired health. The use of TTO may be recommended as it has several favorable methodological qualities. For example, earlier research has shown that it is more consistent with individuals' preferences than other methods such as standard gamble or visual analog scales (3, 4). Implicitly, many decision bodies also recommend the use of TTO valuation, as they, e.g., in the UK and the Netherlands, recommend the use of EQ-5D instruments for measuring and valuing health benefits (5), which rely on TTO tasks (among other methods) for valuation (6–8).

Perhaps unsurprisingly given the widespread use of TTO in practice, a large body of research exists exploring factors that influence individuals' trade-offs between length and quality of life (9). This body of research has, for example, shown that TTO responses depend on individuals' age, sex (10), and religious beliefs (11). Respondents may also consider the consequences of their trade-offs on others, such as family members or children (12–14). Seeing as TTO involves trading off length for quality of life, a large literature also exists studying individuals' willingness to trade of life years, which has shown that such willingness differs systematically between individuals. For example, some individuals assign large importance of being healthy in the near future and are willing to sacrifice many life years for it (15, 16). Furthermore, many respondents are loss averse for health (17, 18), i.e., they assign larger weight to health losses than to health gains. It has been argued that loss aversion yields reluctance to trade-off in TTO [15, (19)]. Individuals may also have different expectations about their remaining life duration, which have been found to affect TTO (20–22). These subjective life expectancies (SLEs) are the focus of this article.

Individuals typically expect to live longer than the duration they are asked to consider in TTO tasks (23, 24). Earlier work has consistently shown that those who expect to live longer are more reluctant to trade-off life duration (20–22). As a result, utilities for those with higher SLEs are typically higher. A potential explanation for this finding in TTO is that individuals may take SLE as their reference point, i.e., they are unwilling to trade-off life duration in TTO because the life on offer falls short from their expectations. Lipman et al. (25) provide a theoretical foundation and empirical support for this claim. Earlier work has found such effects of SLE both in the respondents asked to imagine themselves living in healthy states (21, 22) or patients valuing their current health (20). In this study, it is explored if the effect of SLE also extends to the adults' TTO valuation of child health states, i.e., in the valuation of EQ-5D-Y-3L (26).

EQ-5D-Y-3L is used for the measurement and valuation of health in children aged 8–15 (27). Similar to other EQ-5D instruments used for valuing adult health, it measures health-related quality of life in 5 dimensions: (i) mobility, (ii) self-care, (iii) usual activities, (iv) pain and other complaints, and (v) feeling sad, worried, or depressed. In order to facilitate self-completion by children, the language used in EQ-5D-Y-3L was significantly adapted from the other adult EQ-5D instruments (26, 28). As a result, a separate protocol for the valuation of EQ-5D-Y-3L was developed by the EuroQol group (8). In this protocol, it is recommended that adult respondents value health states considering the life of a 10-year-old child, i.e., they are no longer asked to imagine they themselves live in impaired health but are asked to imagine that the health states affect a child. A series of methodological studies have suggested that the use of such a proxy perspective yields different and typically higher, utilities (29–32).

Thus, the respondents completing TTO typically trade-off fewer years of life for a child than respondents completing TTO for themselves (for the same health state), although the effect differs per health state (29–32). This tendency could have many different explanations (33). For example, individuals may consider the same health state less severe for a child, e.g., because children are better able to adapt to some health impairments (34). It has also been hypothesized that the respondents find children's life years more valuable than their own, e.g., due to differences in time preferences for adults and children (33, 35, 36).

In this article, it is explored if such (un)willingness to trade-off life duration for a child is associated with SLEs. In particular, it can be hypothesized that adult respondents would expect children to have larger remaining life expectancies than themselves, e.g., because they are younger and/or due to generational trends in life expectancy. These differences could provide a potential explanation for higher TTO utilities in proxy perspectives. That is, it has been shown that the temporal distance between SLE and the durations considered in TTO affect willingness to trade, such that increases in this distance yield diminished willingness to trade (25). When adults value EQ-5D-Y-3L with a proxy perspective the distance between the years considered in TTO and their expectations about a 10-year old's longevity will likely be larger than the distance between their own SLE and TTO tasks completed for themselves. If the effect of SLE is the same in both perspectives, this could explain differences in utilities between perspectives for the same health state.

As an illustration, consider the following simple numerical example: imagine a TTO task involving 10 years in some state Q (followed by death), and assume that the utility of that state is known: i.e., adults would sacrifice 5 years to live in full health instead. Now, for simplicity assume that for every 30 years that someone expects to live longer than those 10 years their willingness to trade-off is reduced by 6 months. A 40-year-old respondent expecting to become 70 would sacrifice 4.5 years, whereas if someone would be expected to become 100 they would sacrifice 4 years instead. Now, assume the same logic applies to an adult who values health states with a proxy perspective (i.e., for each 30 years a child falls short of their SLE adults trade-off 6 months fewer). In that case, a TTO task involving 10 years in some state Q for a 10-year-old child, keeping utility of state Q constant, would involve 4 years traded off when adults expect the child to become 70 (distance to SLE: 60 years) and 3.5 years when adults expect the child to become 100 (distance to SLE: 90 years). Ceteris paribus implies higher utilities when using a proxy perspective.

Yet, to date, no studies have explored whether or not EQ-5D-Y-3L valuation is affected by such expectations about the length of life, neither for the adults' expectations about their own length of life nor for expected longevity for a 10-year-old child. In the next sections of this article, the methods and results of a study are reported in which the possibility that EQ-5D-Y-3L valuation is affected by SLE is explored, and rejected. Although respondents generally expected children to become older than themselves, there was no systematic association between their SLE for a child or their own SLE and TTO utilities for EQ-5D-Y-3L valued for a 10-year-old child. In the final sections of the article, potential explanations for this null result are discussed.

## Methods

This study is based on data collected for the Dutch valuation of EQ-5D-Y-3L (37), based on the international protocol developed by Ramos-Goñi et al. (8).

### Sample and Health State Design

A total of 197 respondents were recruited by a marketing agency for taking part in TTO interviews, instructed to ensure a rough balance of gender age and education level. Note that no strict quotas were in place, i.e., the sample might be unbalanced. All the respondents were interviewed by trained TTO interviewers, each of which (*n* = 4) had received a full-day training on EQ-5D instruments and the protocol used for TTO interviewing (7). As is recommended, quality control procedures were implemented to monitor interviewer performance during the study (38). All the interviews were conducted digitally (*via* Zoom), as the Netherlands was in a national lockdown due to the COVID-19 pandemic when data collection for this study was scheduled. That is, respondents were invited for a videotelephony call, with interviewers sharing their screen to guide respondents and entering respondents' answers for TTO tasks into the software designed for this study [for a discussion of the benefits and drawbacks of this approach, see: (39)].

Each respondent valued 10 EQ-5D-Y-3L health states. This version of EQ-5D-Y with 3 answering levels (e.g., no problems, some problems, and a lot of problems) can describe up to 243 unique health states. Note that a version of EQ-5D-Y with 5 answering levels is still in the development (40). As is usual, EQ-5D-Y-3L states are henceforth abbreviated to five-digit codes, such as 11223. This abbreviation would denote a state with no problems with walking about (level 1), no problems with washing and dressing (level 1), some problems with usual activities (level 2), some pain or discomfort (level 2), and feeling very worried, unhappy or sad (level 3). A total of 28 states were included in this study, divided into three blocks. This health state design was adapted from Yang et al. (41). Table 1 shows that each Block contained 9 states with varying severity, and all the blocks contained the worst state captured by EQ-5D-Y-3L, i.e., state 33333. Respondents were randomly assigned to a block.

**Table 1:** EQ-5D-Y-3L health states included per block.

**Block**	**States included**
Block 1	11112, 11121, 11313, 12331, 13133, 31131, 32113, 32322, 33311, 33333
Block 2	11122, 12212, 13221, 21111, 21332, 22222, 23112, 31223, 32232, 33333
Block 3	11211, 12111, 21133, 21211, 21323, 22121, 22233, 23323, 33232, 33333

### Interview Procedure and Measures of SLEs

At the start of the interview, respondents reported their age and sex, as well indicating if they had experience with providing informal care or illness in themselves, family, or friends. Next, in order to become familiar with the levels and dimensions of the instrument, respondents self-completed EQ-5D-Y-3L. In what followed, the procedure described by Stolk et al. (7) was applied to explain the TTO tasks to the respondents. A composite TTO approach was used (42) with a 10-year duration, and a 10-year lead-time for states considered worse than dead. This approach is standard in the valuation of EQ-5D instruments (7, 8). The TTO tasks involved a search procedure (both for states better and worse than dead) for the X (between 0 and 10) number of years in perfect health respondents find equivalent to a life in an impaired health state, denoted *Q*. More details about this search procedure can be found in Stolk et al. (7). The resulting indifference allowed inferring the utility of state Q [i.e., U(Q)], as follows: for states better than dead U(Q) = × /10, for states worse than dead U(Q) = (× -10)/10.

After receiving instructions about the TTO method, each respondent completed 3 practice TTO tasks for states, followed by the 10 states included in the block they were assigned to (in random order). Interviews were concluded by collecting a set of additional demographic information. In this final phase of the interview, two questions were asked that captured individuals' SLE for themselves and for a 10-year-old child. For brevity, the responses to these questions will be referred to as own SLE and child SLE, respectively. Own SLE was measured by asking respondents: “How old do you expect to become?.” Child SLE was measured by the following question: “How old do you think a 10-year-old child will become?.” Respondents were asked to answer in discrete years (e.g., 83 years).

### Data Analysis

Data analysis commenced by reporting and plotting (differences between) own and child SLEs. Next, the association between SLEs and utilities is explored with a set of regression analyses. In contrast to earlier work (21, 22), these analyses are reported with TTO utilities as the dependent variable, instead of years traded. This choice was made, as this study uses composite TTO, which may include lead-time for health states worse than dead. As a result, the same number of years traded would have a different implication. By using utilities as the dependent variables, we can interpret the effect of SLEs on TTO trade-offs regardless of the method used. Furthermore, compared with the earlier work (21, 22) more states were included in this study. The influence of SLEs across states is tested by controlling for health state severity through a level-sum-score (LSS). This LSS is calculated as the sum of all EQ-5D-Y-3L levels. That is, state 11111 has an LSS of 5, and 33333 has an LSS of 15. Hence, a set of mixed-effects ordinary least squares (OLS) regressions was run with subject random effects and fixed effects for: (i) health state severity (LSS), (ii) own SLE, (iii) child SLE, (iv) age, (v) sex (reference: female), and (vi) parental status (reference: no kids). To explore if the effect of SLE on TTO utilities differed between health states considered better than dead or worse than dead this model was ran on three times: (i) for all TTO data, (ii) for all TTO utilities for states better than or equal to dead (i.e., U(Q) ≥ 0), and (iii) for all TTO utilities for worse than dead states [i.e., U(Q) < 0]. The results of these overall analyses are further substantiated by analyses per health state (28 in total). Seeing as this study is of exploratory nature, no correction for multiple hypothesis testing is applied (and given the null result reported such correction would not affect the conclusions of the study).

## Results

In this study, the results reported focus on the effects of SLE on TTO valuation for EQ-5D-Y-3L. Note that the study was designed for constructing a value set for EQ-5D-Y-3L, but such a value set, as well as the descriptive results for all the health states (e.g., mean utilities), will not be reported here to avoid confusion and/or overlap in subsequent publications in which the Dutch value set for EQ-5D-Y-3L will be reported (37).

### Sample Descriptive Statistics

Table 2 shows descriptive statistics for the sample recruited for this study, as well as presenting frequency statistics for the respondents' self-completed EQ-5D-Y-3L. As expected, the sample appears to be non-representative, as females and highly educated individuals appeared to be oversampled. The respondents' mean age was lower than that of the Dutch adult population, which is ~49.6 (43). Nonetheless, a reasonable spread in age was obtained, with the minimum, first quartile, median, third quartile, and maximum ages of respondents being: 18, 29, 40, 52, and 75, respectively. Furthermore, the sample generally does not self-report health problems, with some pain or discomfort being one of the problems most likely to be reported. The three most reported EQ-5D-Y-3L health states were: 11111 (*n* = 105), 11121 (*n* = 23), and 11112 (*n* = 17).

**Table 2:** Descriptive statistics and self-reported health for full sample (*n* = 197).

	**Full sample (*n* = 197)**		**Full sample (*n* = 197)**
**Age—M (SD)**	41.87 (14.26)	**EQ-5D-Y-3L**	
**Sex—*****N*** **(%)**		**Mobility—*****N*** **(%)**	
Males	84 (42.6%)	No problems	180 (91.4%)
Females	113 (57.4%)	Some problems	13 (6.6%)
**Marital status—*****N*** **(%)**		A lot of problems	4 (2.0%)
Married/Registered partners	113 (57.4%)	**Self-care—*****N*** **(%)**	
Single	72 (36.5%)	No problems	196 (99.5%)
Divorced	9 (4.6%)	Some problems	1 (0.5%)
Widowed	3 (1.5%)	A lot of problems	0 (0%)
**Parental status—*****N*** **(%)**		**Usual activities—*****N*** **(%)**	
Parents	92 (46.7%)	No problems	165 (83.8%)
**Education level—*****N*** **(%)**		Some problems	28 (14.2%)
Lower education	12 (6.1%)	A lot of problems	4 (2.0%)
Higher education	63 (32.0%)	**Pain/Discomfort—*****N*** **(%)**	
University education	122 (61.9%)	No pain or discomfort	135 (68.5%)
**Income—*****N*** **(%)**		Some pain or discomfort	59 (29.9%)
< € 14.000	17 (8.6%)	A lot of pain or discomfort	3 (1.5%)
€14.000-€27.999	22 (11.2%)	**Anxiety/Depression—*****N*** **(%)**	
€28.000-€41.999	34 (17.3%)	Not sad, worried, or unhappy	150 (76.1%)
€42.000-€55.999	30 (15.2%)	A bit sad, worried, or unhappy	46 (23.4%)
€56.000-€69.999	23 (11.7%)	Very sad, worried, or unhappy	1 (0.5%)
€70.000-€90.999	16 (8.1%)		
>€91.000	13 (6.6%)	**EQ-VAS—M (SD)**	81.55 (11.23)

### Own and Child SLE

**Figure 1:**
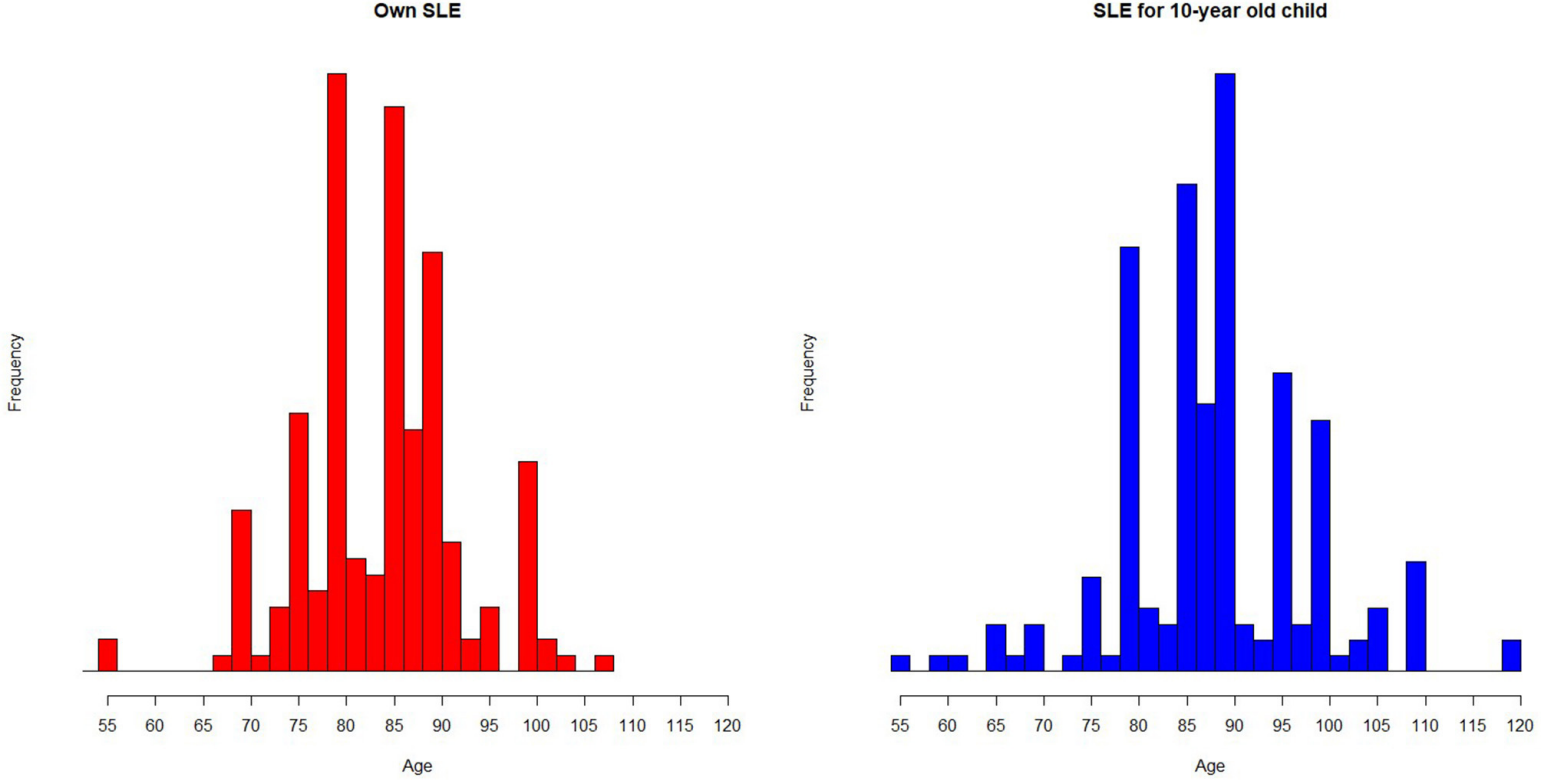
Histogram depicting distribution of own and child SLE.

One respondent reported expecting to become 0 years old. This outlier response was treated as missing in the remaining analyses. The distribution of the remaining respondents' own SLE and child SLE is seen in Figure 1. Respondents' own mean SLE was 83.8 (SD = 10.3), which entailed their expected remaining life duration was 42.3 (SD = 16.70) years. There was no correlation between age and own SLE (*p* = 0.76). Child SLE was 88.47 (SD = 10.11) on average, which is significantly higher than own SLE [*t*_(195)_ = 6.01, *p* < 0.001].

**Figure 2:**
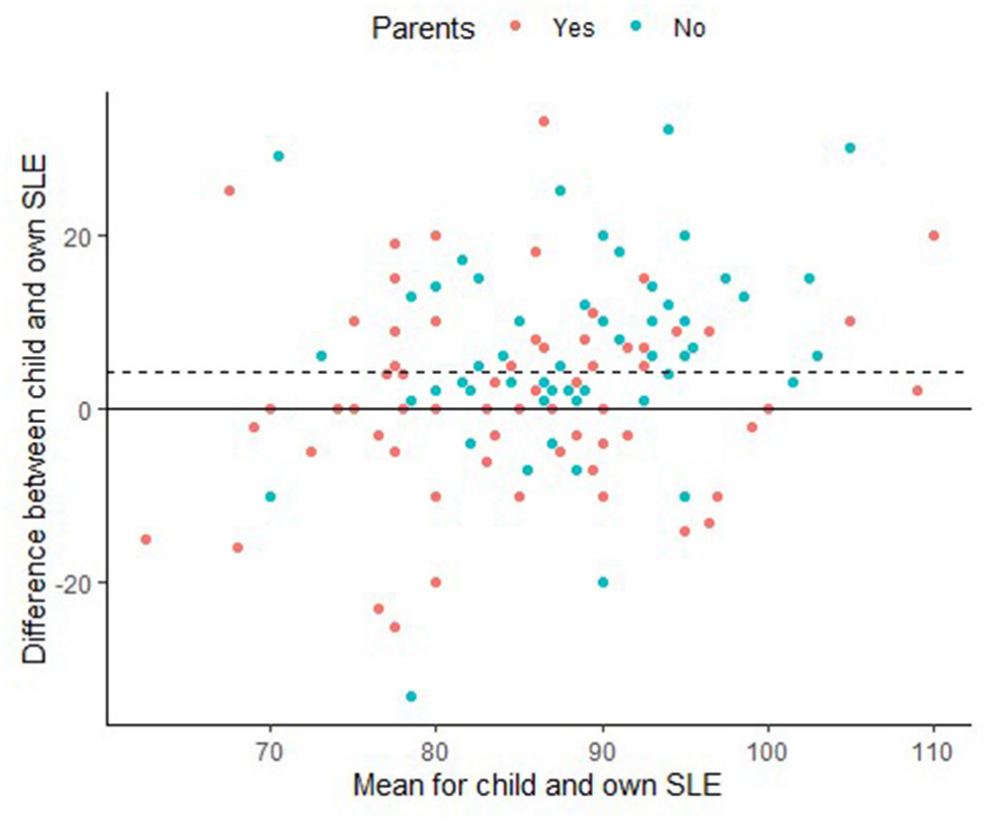
Bland-Altman plot showing within subjects differences between child and own SLE (dashed line indicates mean difference).

Figure 2 shows the within-subject difference between own and child SLE in a Bland-Altman plot. In this plot, the average SLE (i.e., average of own and child SLE) is plotted against the difference between own and child SLE. Surprisingly, a trend was observed that suggested parents expect children to die at an earlier age (*M* = 87.2, SD = 10.7), than respondents without children (*M* = 89.8, SD = 9.4), *t*_(194)_ = −1.76, *p* = 0.08). To accommodate different expectations between parents and non-parents, colors are used to indicate parental status. As can be seen from Figure 2, the mean difference between own and child SLE was positive, reflecting that a majority of respondents (*n* = 130) expect a 10-year-old child to become older than themselves. Nonetheless, the opposite expectation (i.e., own SLE > child SLE) also occurred for 37 respondents, and 29 respondents expected to become exactly as old as a 10-year-old child. Although own and child SLE were moderately correlated [*r*_(194)_ = 0.43, *p* < 0.001], the differences between both expectations were not correlated with the respondents' age (*p* = 0.85).

### Association Between Utilities and SLEs

Table 3 reports the OLS regressions, where Model 1 reports the results for all utilities, and Models 2 and 3 include only health states valued better (or equal) to dead and worse than dead health states, respectively. As can be seen from Table 3, when all data were included, there was no overall association between either SLE and utilities, or any of the other demographics. In fact, only health state severity predicts utilities. Severity was the strongest predictor across all the three models. If only utilities better or equal to dead are included, a marginally significant association between child SLE and utilities is found, which suggests a trend of higher utilities for those that expect children to die older. For utilities worse than dead, no such effect is found, but an effect of age is observed, which suggests that those that are older have more strongly negative utilities. Note that this null result for the effect of SLE on EQ-5D-Y-3L is robust to different specifications. That is, none of the conclusions for SLE are affected by: (i) only including child SLE or own SLE, (ii) including a child and/or own SLE in the regression models as dichotomous variables after performing a median split, (iii) specifying the models *remaining* subjective life expectancy, i.e., own remaining SLE = own SLE–age and child remaining SLE = child SLE-−10, (iv) replacing both SLE predictors in Models 1–3 by a single predictor that reflects the difference between own SLE and child SLE, and (v) replacing both SLE predictors by a single predictor reflecting the difference between own remaining SLE and child remaining SLE.

**Table 3:** OLS regression results for mixed effects regression on EQ-5D-Y-3L utilities including effects of own and child SLE.

	**Model 1: all data**	**Model 2: better than dead**	**Model 3: worse than dead**
Constant	1.726 (0.256)^***^	1.121 (0.117)^***^	−0.524 (0.251)^*^
Severity: LSS	−0.134 (0.004)^***^	−0.065 (0.002)^***^	−0.019 (0.006)^***^
Own SLE	0.004 (0.003)	0 (0.001)	0.001 (0.003)
Child SLE	−0.002 (0.002)	0.002 (0.001)+	0.001 (0.002)
Age	−0.002 (0.002)	0.001 (0.001)	−0.005 (0.002)^**^
Sex: Male	0.063 (0.046)	0.016 (0.021)	0.078 (0.048)
Parental status: parents	−0.022 (0.052)	0.012 (0.024)	−0.043 (0.051)
*n*	*1,960*	*1,639*	*321*

+, ^*^, ^**^, and ^***^ represent significance at p < 0.10, p < 0.05, 0.01, and 0.001, respectively.

### Association Between SLE and Utilities per Health State

To further substantiate the (lack of) systematic associations between SLEs and EQ-5D-Y-3L utilities, a subsequent set of OLS regressions was performed for each health state. Given that for each state only a single response was captured per respondent, no random effects were included. Otherwise, the regressions included the same predictors as Models 1–3. Table 4 shows the OLS regression results.

**Table 4:** OLS regression estimates (standard error in brackets) per health state.

	***N***	**Constant**	**Own SLE**	**Child SLE**	**Age**	**Parental status: parents**	**Sex: male**
11112	64	1.125 (0.368)^**^	−0.004 (0.004)	0.001 (0.004)	0 (0.003)	0.102 (0.076)	−0.04 (0.085)
11121	64	1.107 (0.121)^***^	0 (0.001)	−0.001 (0.001)	0 (0.001)	0.031 (0.025)	−0.007 (0.028)
11122	66	0.776 (0.283)^**^	0.002 (0.003)	0 (0.002)	−0.002 (0.002)	−0.074 (0.042)	0.105 (0.046)^*^
11211	66	0.753 (0.099)^***^	0 (0.001)	0.003 (0.001)^*^	0 (0.001)	0.006 (0.019)	0.007 (0.022)
11313	64	0.48 (0.905)	0.004 (0.011)	−0.008 (0.01)	0.01 (0.008)	0.05 (0.186)	−0.342 (0.209)
12111	66	0.912 (0.067)^***^	−0.002 (0.001)^*^	0.003 (0.001)^***^	0 (0)	−0.014 (0.013)	0.024 (0.015)
12212	66	0.839 (0.29)^**^	0.001 (0.003)	−0.001 (0.002)	0 (0.002)	−0.041 (0.043)	0.03 (0.048)
12331	64	0.261 (0.886)	0.001 (0.01)	0.002 (0.01)	−0.002 (0.008)	−0.032 (0.182)	0.014 (0.205)
13133	64	−0.582 (0.964)	0.009 (0.011)	−0.001 (0.011)	−0.001 (0.008)	−0.049 (0.198)	−0.085 (0.222)
13221	66	0.746 (0.316)^*^	0.002 (0.003)	−0.001 (0.003)	−0.001 (0.002)	−0.049 (0.047)	0.066 (0.052)
21111	66	1.015 (0.199)^***^	0 (0.002)	0 (0.002)	−0.002 (0.001)	−0.042 (0.029)	0.087 (0.033)^**^
21133	66	0.785 (0.863)	−0.002 (0.012)	−0.001 (0.008)	−0.014 (0.006)^*^	0.191 (0.162)	0.024 (0.193)
21211	66	0.56 (0.201)^**^	−0.001 (0.003)	0.005 (0.002)^**^	0 (0.001)	−0.005 (0.038)	0.013 (0.045)
21323	66	−0.044 (0.752)	0 (0.01)	0.006 (0.007)	−0.006 (0.005)	0.167 (0.141)	0.073 (0.168)
21332	66	1.784 (1.017)	−0.007 (0.01)	−0.008 (0.009)	0 (0.006)	0.15 (0.151)	−0.242 (0.167)
22121	66	0.553 (0.207)^**^	−0.001 (0.003)	0.004 (0.002)^*^	0.001 (0.001)	0.025 (0.039)	0.015 (0.046)
22222	66	0.942 (0.395)^*^	−0.002 (0.004)	0 (0.003)	−0.001 (0.002)	−0.007 (0.059)	0.044 (0.065)
22233	66	−0.659 (0.831)	0.017 (0.011)	−0.005 (0.008)	−0.012 (0.006)	0.243 (0.156)	0.137 (0.186)
23112	66	0.757 (0.251)^**^	0.003 (0.002)	−0.003 (0.002)	0 (0.002)	−0.053 (0.037)	0.097 (0.041)^*^
23323	66	−0.178 (0.78)	0.01 (0.01)	−0.003 (0.008)	−0.008 (0.006)	0.31 (0.147)^*^	−0.006 (0.174)
31131	64	0.256 (0.875)	0.006 (0.01)	−0.005 (0.01)	0.006 (0.008)	0.043 (0.18)	−0.173 (0.202)
31223	66	0.823 (1.016)	0.002 (0.01)	−0.008 (0.009)	0.001 (0.006)	0.175 (0.151)	−0.115 (0.167)
32113	64	−0.389 (0.839)	0.002 (0.01)	0.007 (0.009)	0.003 (0.007)	−0.019 (0.173)	−0.284 (0.194)
32232	66	0.363 (1.046)	0.015 (0.01)	−0.013 (0.009)	−0.002 (0.007)	0.104 (0.155)	−0.149 (0.172)
32322	64	0.064 (0.53)	0.008 (0.006)	−0.003 (0.006)	0.006 (0.005)	−0.04 (0.109)	−0.181 (0.122)
33232	66	−0.02 (0.79)	0.01 (0.011)	−0.001 (0.008)	−0.011 (0.006)	−0.074 (0.148)	0 (0.176)
33311	64	1.096 (0.502)^*^	0.002 (0.006)	−0.005 (0.006)	−0.001 (0.004)	0.077 (0.103)	−0.113 (0.116)
33333	196	−0.496 (0.541)	0.003 (0.006)	−0.001 (0.005)	−0.005 (0.004)	0.209 (0.099)^*^	0.112 (0.112)

Significant coefficients (for all but the constants) are printed in bold, where ^*^, ^**^, and ^***^ represent significance at p < 0.05, 0.01, and 0.001, respectively. These p–values are not corrected for multiple testing.

As can be seen from Table 4, only some evidence is observed in support of the effects of SLEs on EQ-5D-Y-3L utilities. That is, a significant negative association between own SLE and utility for state 12111 was observed, suggesting that for this single state (out of 28) individuals who expect to become older themselves value this state lower. Associations in the opposite direction are observed for child SLE and the following 4 (out of 28) health states: 11121, 12111, 21211, and 22121. These per-state analyses also yield several associations between demographics and EQ-5D-Y-3L utilities. Older respondents had significantly lower utilities for state 21133, parents had higher utilities for states 23323 and 33333, and males had higher utilities for states 11122, 21111, and 23112. However, given that no correction for multiple hypothesis testing is applied, these results should be interpreted with caution. These analyses suggest that the effects of SLEs and demographics are non-systematic, as also suggested in Table 3. As a further illustration of this non-systematicity, consider, for example, that the sign of the regression coefficient for own SLE (child SLE) was positive for 21 (12) states and negative for the remaining 7 (22) states. Hence, the significant effects observed in Table 4 are in a direction opposite to the most occurring direction.

## Discussion

This study explored the effect of subjective expectations about the children's and adults' length of life on EQ-5D-Y-3L valuation with a proxy perspective. Such exploration was considered relevant, as it was hypothesized that individuals expect children to become older than themselves, and earlier work has shown that such higher SLE yields reluctance to trade-off life duration (20–22, 25). As such, differences between own SLE and child SLE may partly explain earlier research that found that individuals' trade-off fewer life years when valuing health with proxy perspectives (29, 31, 32, 44). The results of this article, however, suggest that although individuals expect children to become older than themselves, neither own nor child SLE is associated with EQ-5D-Y-3L utilities. Hence, no (or perhaps very little) evidence is found in favor of the hypothesis that motivated this study, i.e., that SLE may explain higher TTO utilities when valuing child health. A lack of evidence as observed in this study can either mean that no effect exists, or alternatively that the limitations of this study precluded observing a “true effect.” Both options are discussed in turn.

When interpreting the lack of evidence observed in this study as evidence of no effect, a finding in contrast to earlier health state valuation research is observed. That is, earlier published work has consistently found a positive association between SLE and willingness to trade-off life duration in TTO (20–22). The findings of this study can be interpreted as suggesting that no such association exists when adults trade-off life duration for a 10-year-old child. Interestingly, TTO responses are neither associated with their own SLE or their expectations about the longevity of a 10-year-old child. Thus, although Lipman et al. (25) suggested SLE can serve as a reference point in TTO valuation, such expectations are not taken as reference-point with proxy perspectives. Potentially, this lack of external reference points could be explained by the psychological distance introduced through the use of a proxy perspective, as modeled in Construal Level Theory (45).

Yet, this study differed on more than just the perspective (i.e., proxy instead of an adult) to earlier studies that observed an association between SLE and TTO utilities. Whereas, this study used a relatively small sample valuing a large number of health states, most published studies would have the opposite characteristic (large sample, few health states). Furthermore, some studies involved TTO tasks that respondents completed without supervision (21, 22, 46), whereas the presence of interviewers (47) and the use of a quality control process (38) have been found essential to ensure TTO responses of sufficient quality. Potentially, individuals incorporating their views on life duration beyond the projected short durations considered in TTO are engaging in the low-quality trade-offs that interviewers and a quality control process preclude. Hence, a recommendation is to explore if the effects of SLE observed in earlier work are a byproduct of using suboptimal TTO valuation methods. In particular, further research aiming to substantiate or replicate these results should use a design in which questions about own and child SLE are included both for adults valuing hypothetical health states for themselves, as well as valuing EQ-5D states with a proxy perspective. A study with this design would allow a full test of the hypothesis that motivated this study.

Nonetheless, it cannot be excluded that the limitations of this study led to an incorrect rejection of this study's hypothesis. At least the following limitations deserve mentioning. First, due to the COVID-19 pandemic study teams may have been necessitated to use videotelephony TTO valuation. At this stage, there is no evidence that proves the formal equivalence of interviews performed in person and *via* software packages such as Zoom, although there is some evidence suggesting adequate performance of videotelephony (39, 48). Second, although the sample of this study was more diverse than some earlier work studying SLE (25), females and those with higher education were oversampled. Earlier work has suggested that both may affect EQ-5D valuation (9, 10), i.e., subsequent studies investigating the influence of SLE on TTO valuation with proxy perspectives may recruit representative samples. Third, our study collected information about SLE after TTO valuation, whereas some earlier work collected information on SLE before TTO valuation (20, 25). Although potentially prompting respondents to consider life expectancies beyond the short durations used in TTO, this ordering used in earlier research avoids respondent fatigue (which is often assumed to be substantial for TTO) affecting estimates for SLE. If respondent fatigue increased noise in the estimates for own and child SLE in this study, the relatively small sample size used may have led to an underpowered study. Furthermore, the use of a proxy perspective asks adult respondents to think of life and death for a 10-year-old child, which has been found to be complex and at times invoking negative emotions in think-out-loud research (34). It is possible that going through this process could have a systematic influence on child SLE, which may be explored in the future research.

In conclusion, it appears that, in contrast to findings in adult health state valuation, expectations about the length of life are not a predictor of willingness to trade-off life duration in EQ-5D-Y valuation with a proxy perspective.

## Data Availability Statement

The datasets presented in this article are not readily available because the data is proprietary. Requests to access the datasets should be directed to https://euroqol.org/.

## Ethics Statement

The studies involving human participants were reviewed and approved by Erasmus School of Health Policy & Management's Internal Review Board. The patients/participants provided their written informed consent to participate in this study.

## Author Contributions

SL: study conception and design, analysis and interpretation of results, and manuscript preparation.

## Funding

Funding the data collection for this study was provided by the EuroQol Research Foundation (grant number: 100-2020VS).

## Author Disclaimer

The views expressed by the author do not necessarily reflect the views of the EuroQol Group.

## Conflict of Interest

SL has received funding from the EuroQol Research Foundation for research outside the current article.

## Publisher's Note

All claims expressed in this article are solely those of the authors and do not necessarily represent those of their affiliated organizations, or those of the publisher, the editors and the reviewers. Any product that may be evaluated in this article, or claim that may be made by its manufacturer, is not guaranteed or endorsed by the publisher.
